# An Improved HRNetV2-Based Semantic Segmentation Algorithm for Pipe Corrosion Detection in Smart City Drainage Networks

**DOI:** 10.3390/jimaging11100325

**Published:** 2025-09-23

**Authors:** Liang Gao, Xinxin Huang, Wanling Si, Feng Yang, Xu Qiao, Yaru Zhu, Tingyang Fu, Jianshe Zhao

**Affiliations:** School of Artificial Intelligence, China University of Mining & Technology, Beijing 100083, China; xxhuang0624@163.com (X.H.); sqt2310405030@student.cumtb.edu.cn (W.S.); qiaoxu@cumtb.edu.cn (X.Q.); zqt2410405054@student.cumtb.edu.cn (Y.Z.); sqt2310405023@student.cumtb.edu.cn (T.F.); zqt2310405045@student.cumtb.edu.cn (J.Z.)

**Keywords:** semantic segmentation, urban drainage pipeline, HRNetV2, CBAM, pyramid pooling, corrosion detection, smart city, smart infrastructure, deep learning

## Abstract

Urban drainage pipelines are essential components of smart city infrastructure, supporting the safe and sustainable operation of underground systems. However, internal corrosion in pipelines poses significant risks to structural stability and public safety. In this study, we propose an enhanced semantic segmentation framework based on High-Resolution Network Version 2 (HRNetV2) to accurately identify corroded regions in Traditional closed-circuit television (CCTV) images. The proposed method integrates a Convolutional Block Attention Module (CBAM) to strengthen the feature representation of corrosion patterns and introduces a Lightweight Pyramid Pooling Module (LitePPM) to improve multi-scale context modeling. By preserving high-resolution details through HRNetV2’s parallel architecture, the model achieves precise and robust segmentation performance. Experiments on a real-world corrosion dataset show that our approach attains a mean Intersection over Union (mIoU) of 95.92 ± 0.03%, Recall of 97.01 ± 0.02%, and an overall Accuracy of 98.54%. These results demonstrate the method’s effectiveness in supporting intelligent infrastructure inspection and provide technical insights for advancing automated maintenance systems in smart cities.

## 1. Introduction

Urban drainage networks form a vital component of smart city infrastructure, re-sponsible for managing stormwater and sewage to sustain urban water cycles and ensure public safety. The structural integrity of these underground pipelines directly impacts ur-ban flood control and environmental health [1]. Due to long-term operation, these pipelines suffer from continuous water flow erosion, internal pressure, corrosive environmental influences [2], and adverse factors such as ground settlement during urban construction, leading to a wide range of structural damages, including corrosion, cracks, and deformation [3]. Among them, internal corrosion is a typical structural defect that gradually erodes the pipe wall material and is a major cause of leakage, rupture, and even collapse. Traditional closed-circuit television (CCTV) inspection relies heavily on manual interpretation of videos by professionals, which suffers from inefficiency, subjectivity, and difficulty in quantitative assessment [4,5]. These limitations hinder the intelligent operation and maintenance of infrastructure required by smart cities. Therefore, the development of high-precision, automated corrosion detection technologies has become a central challenge in smart water management, with significant implications for ensuring underground spatial safety.

Image segmentation, a fundamental task in computer vision, aims to partition images into regions with specific semantics or common features, serving as a foundation for higher-level tasks such as object recognition and scene understanding. Conventional image segmentation methods based on low-level visual features (e.g., grayscale, color, texture) include thresholding, edge detection, clustering, region growing, and graph-based methods. These approaches have shown effectiveness in constrained scenarios. For example, edge detection methods like the classic Canny operator attempt to delineate object boundaries by identifying abrupt changes in intensity or texture [6]; morphological operations based on pseudo top-hat transforms have been applied to enhance dark-region boundaries and extract pipe defects of specific shapes [7,8,9]; dynamic thresholding segments images by pixel intensity [10]; graph-based methods model the image as a graph and perform segmentation using graph cuts or partitioning algorithms, applied in water leakage detection [11] and image partitioning [12]; and clustering methods like K-Means group pixels based on similarity in feature space, with applications in corrosion image analysis [13,14]. However, such methods often rely on hand-crafted feature engineering, prior knowledge, and sensitive parameter tuning, making them vulnerable to noise, illumination variation, complex backgrounds, and the diverse morphology of defects. Thresholding is sensitive to uneven lighting; edge detection struggles with noise and incomplete boundaries; region growing depends on seed point selection and growth criteria; and graph-based methods may suffer from high computational complexity and difficult parameterization [12,15].

In recent years, the rapid advancement of deep learning, particularly Convolutional Neural Networks (CNNs), has brought a paradigm shift to the field of image segmentation. Semantic segmentation, which assigns a semantic label to every pixel in an image, has emerged as a foundational technique for pixel-level image understanding. Its ability to precisely delineate object shapes and locations has demonstrated great potential in critical applications such as infrastructure health monitoring and industrial defect inspection [16]. Compared to object detection, semantic segmentation provides pixel-wise granularity, offering a solid technical foundation for subsequent quantitative defect evaluation [17]. Significant progress has been made in applying deep learning–based semantic segmentation to surface defect detection in pipelines and bridge structures. Encoder–decoder architectures, such as U-shaped convolutional networks (U-Net) and its variants, have become mainstream solutions due to their excellent feature fusion capabilities. U-Net, proposed by Ronneberger et al. [18], introduced skip connections to effectively fuse high- and low-level features and has since become a leading framework for pipe defect detection. Subsequent works have further improved performance by integrating attention mechanisms such as the Convolutional Block Attention Module (CBAM), Coordinate Attention (CA), and Squeeze-and-Excitation (SE), enabling networks to focus more effectively on defect regions while suppressing interference from complex pipeline backgrounds [19,20]. Models such as DeepLabv3+, SparseInst, and instance segmentation architectures have been employed to achieve more refined localization and differentiation of defects [21]. Liu et al. [22] enhanced DeepLabv3+ using Atrous Spatial Pyramid Pooling (ASPP) to improve multi-scale defect recognition, incorporating Efficient Channel Attention (ECA) modules in the encoder–decoder to focus on critical information, thereby boosting feature representation and segmentation accuracy. Wang et al. [23] designed the SparseInst framework for high-precision instance-level defect segmentation, introducing TensorRT acceleration to meet real-time inspection demands. Moreover, ensemble learning strategies, data augmentation via Generative Adversarial Networks, multimodal fusion, and Conditional Random Field (CRF)–based post-processing have been extensively explored to improve robustness, generalization, and boundary precision. Forkan et al. [24] proposed CorrDetector, employing ensemble learning for Unmanned Aerial Vehicle (UAV) corrosion detection with strong resilience to noise and large-scale variations. Li et al. [25] leveraged Style-Based Generative Adversarial Network 3 (StyleGAN3) to synthesize corrosion images for addressing data scarcity and designed an enhanced DeepLabv3+ to achieve improved segmentation under few-shot conditions. Papamarkou et al. [26] applied residual networks to automatically identify corrosion in dry nuclear fuel storage casks, combining preprocessing and residual connections to boost detection accuracy with minimal manual annotation. Jin et al. [27] integrated sonar and optical imaging for multimodal underwater defect recognition and guided the network using channel and spatial attention to enhance multi-defect identification, particularly in blurry and low-contrast sonar images. Wang [28] incorporated CRF to refine segmentation boundaries, improving contour accuracy and consistency, especially for corrosion-crack hybrid defect scenarios.

The core advantage of deep learning lies in its end-to-end feature learning capability, enabling it to learn highly discriminative hierarchical features directly from raw data, thus overcoming the limitations of manually crafted features in traditional methods [29,30]. Numerous studies have confirmed that deep learning models significantly outperform traditional approaches in terms of segmentation accuracy, robustness, and generalization across challenging scenarios involving illumination variations, noise, cluttered backgrounds, and morphological diversity of pipeline defects. These models have been successfully applied in UAV inspection, industrial endoscopy, CCTV-based sewer inspection [31], and nuclear facility monitoring. For instance, Nash et al. [32] constructed a corrosion annotation dataset through crowdsourcing and used CNNs for automated detection of multiple corrosion types in industrial settings, reducing annotation cost and enhancing model generalization. Subsequently, Burton et al. [33] proposed the RustSEG semantic segmentation framework, combining U-Net and Residual Network (ResNet) encoders for pixel-wise corrosion segmentation. Their model achieved an up to 85% mean Intersection over Union (mIoU) even under oil contamination and severe rust interference, effectively addressing challenges such as fuzzy boundaries and small defect detection, demonstrating strong practical value. Despite these advances, deploying deep-learning-based semantic segmentation in real-world urban drainage pipeline inspection remains challenging. Firstly, obtaining a large number of high-quality, pixel-level labeled defect images is costly and labor-intensive, limiting model training. Secondly, complex background elements—such as pipe textures, water residue, sludge deposits, and structural shadows—often resemble real defects, leading to false positives and missed detections, requiring models to have stronger contextual reasoning and anti-interference capabilities. Thirdly, fine-grained defects (e.g., cracks, pitting, patchy corrosion) exhibit diverse scales and shapes, demanding models with effective multi-scale feature extraction capabilities. However, traditional U-Net models suffer from high-frequency detail loss during downsampling, resulting in blurred boundaries. DeepLabv3+–like networks often destroy local textures due to dilated convolutions. Furthermore, segmentation performance may vary under different pipe materials, aging conditions, imaging devices, or environmental settings, revealing limitations in model robustness and transferability.

Despite progress, three major challenges persist in applying deep learning to drainage pipe corrosion segmentation. Firstly, Weak boundaries and irregular shapes of corrosion regions often lead to false negatives or blurred edges. Secondly, Complex and noisy backgrounds (e.g., water residues, shadows) cause false positives, reducing reliability in real inspection conditions. Finally, Multi-scale variability in corrosion (from small pits to large eroded patches) demands effective context modeling, which existing methods inadequately capture.

To address the above challenges, especially the difficulty of detecting small-scale corrosion in complex backgrounds and the risk of background-induced false detections, this study proposes an improved semantic segmentation framework based on the High-Resolution Network Version 2 (HRNetV2). The main contributions of this paper are summarized as follows:

1. An improved HRNetV2 architecture tailored for precise segmentation of corrosion regions in complex drainage pipe environments is proposed. For the first time, this framework systematically integrates the CBAM and a Lightweight Pyramid Pooling Module (LitePPM), jointly improving the network’s ability to perceive multi-scale defects and resist background interference.
2. Inspired by the Pyramid Scene Parsing Network (PSPNet), a LitePPM is designed. This module uses adaptive average pooling at multiple scales, followed by convolution and feature concatenation to extract and fuse contextual information, expanding the network’s receptive field while controlling parameter growth.
3. Comprehensive experiments on a self-built drainage pipe defect dataset validate the effectiveness of the proposed approach. Results show that the model significantly outperforms mainstream U-Net variants and the original HRNetV2 in terms of segmentation accuracy, mIoU and Recall.

## 2. Materials and Methods

### 2.1. Drainage Pipeline Data Acquisition

**Figure 1:**
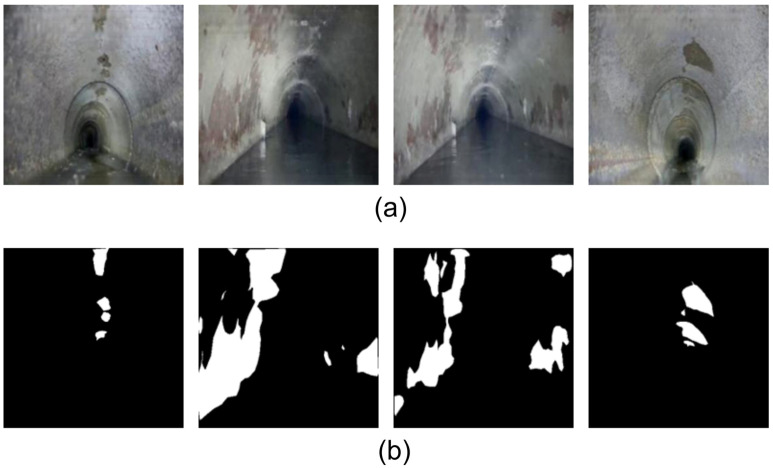
Original and annotated images of partial pipeline corrosion: (**a**) Original images of corrosion defects in drainage pipelines; (**b**) Annotated label images, where white regions represent corrosion areas and black regions represent the background.

The detailed description of the dataset is summarized in Table 1. The dataset used in this study originates from the corrosion images of drainage pipelines captured by a CCTV pipe inspection robot developed at China University of Mining & Technology, Beijing, China. The original images were screened by professionals to ensure quality. These images cover a diverse range of viewpoints, including front views, side views, and partial views of the pipeline, totaling 1360 corrosion images. This dataset is highly suitable for corrosion area segmentation tasks in drainage pipelines. Pixel-level annotations were carried out using the LabelMe software version 5.0.1, where the corrosion regions were labeled as “FS.” The annotated data was then converted into the VOC dataset format. Finally, the dataset was divided into training, validation, and testing sets at a ratio of 8:1:1. Figure 1a presents examples of the original images showing corrosion defects in drainage pipelines, and Figure 1b shows the corresponding annotated label images, where black represents the background and white indicates the corroded regions.

### 2.2. Data Augmentation

**Figure 2:**
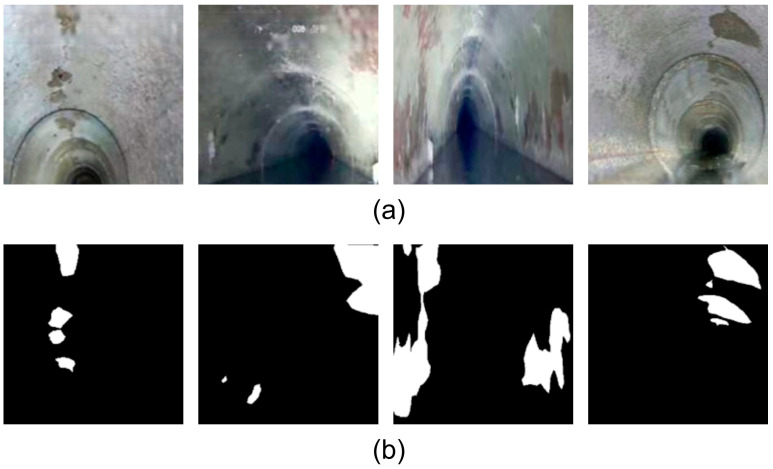
Enhanced original and annotated images of partial pipeline corrosion: (**a**) Enhanced original images; (**b**) Enhanced annotated label images, where white regions represent corrosion areas and black regions represent the background.

To improve the model’s generalization ability in the corrosion area segmentation task for drainage pipelines, a variety of data augmentation strategies were employed during the data loading stage. These include random scaling and cropping, horizontal flipping, Gaussian blurring, random rotation, and color distortion. These augmentation techniques are applied in random combinations to each pair of input image and label, which effectively increases the diversity of training samples and reduces the risk of model overfitting. These techniques are highly controllable, flexible, and computationally efficient with low implementation complexity. Examples of the augmented corrosion area images and their corresponding labels are shown in Figure 2.

### 2.3. Semantic Segmentation Network for Corrosion Areas in Drainage Pipelines

**Figure 3:**
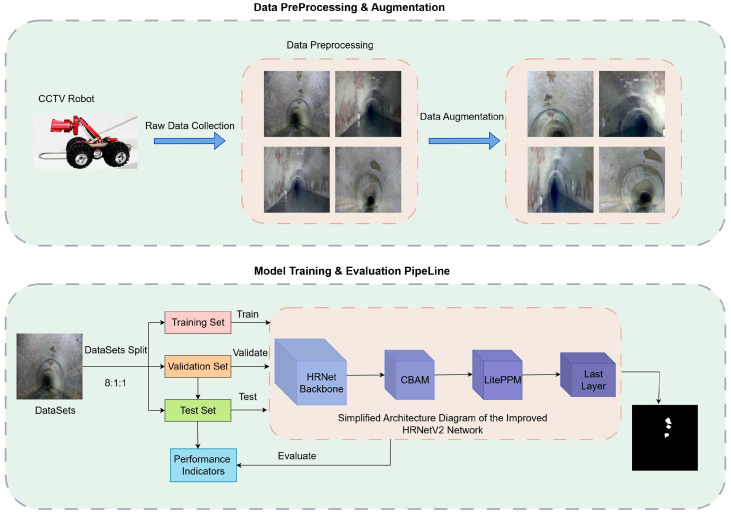
Workflow of the proposed method.

To address the challenges encountered by traditional networks in such segmentation tasks, this study proposes a multi-branch high-resolution parallel network based on the HRNetV2 architecture. The framework of the proposed method is shown in Figure 3. First, raw image data of internal sewer defects are acquired using the CCTV inspection robot. The collected images are then preprocessed by resizing them and padding gray borders to match the network input dimensions. Based on this, data augmentation techniques such as random scaling, horizontal flipping, translation cropping, Gaussian blurring, random rotation, and color disturbance are applied to further enrich the dataset and enhance the model’s generalization. The dataset is divided into training, validation, and testing sets in an 8:1:1 ratio. This study modifies the baseline HRNetV2 by integrating the CBAM attention mechanism to enhance the feature response of key areas and embeds the LitePPM module during high-level semantic feature extraction to achieve multi-scale information fusion and aggregation. Finally, the predicted segmentation maps output by the model enable pixel-level identification of corroded regions within the sewer. Model performance is validated and analyzed using various evaluation metrics on the test set.

#### 2.3.1. HRNetV2 Semantic Segmentation Model

High-Resolution Network (HRNet) was first proposed by Sun et al. [34] at CVPR 2019. Its core innovation lies in breaking away from the traditional encoder–decoder structure by constructing parallel multi-resolution subnetworks that maintain high-resolution representations throughout the network. The HRNet series includes High-Resolution Network Version 1 (HRNetV1), HRNetV2, High-Resolution Network Version 2 Plus (HRNetV2P). HRNetV1 outputs only high-resolution branch features, lacking deep semantic utilization. Later that year, Sun et al. proposed HRNetV2 and HRNetV2P for segmentation and detection tasks, respectively [35]. HRNetV2 fuses deep semantic features from all branches while preserving details and providing global semantic context information.

**Figure 4:**
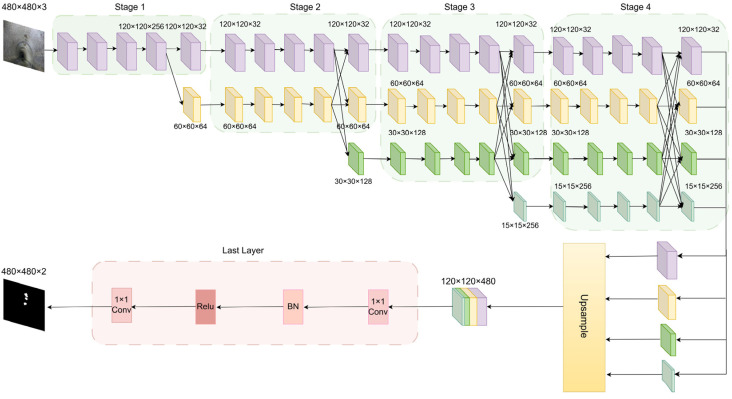
Network architecture of the HRNetV2 model.

The network structure of the HRNetV2 semantic segmentation model is illustrated in Figure 4. It consists of three parts: backbone feature extraction, feature integration, and prediction output. The backbone includes four stages. The input image size is 480 × 480 × 3. In the first stage, two 3 × 3 convolutions with a stride of 2 downsample the image to 120 × 120 and increase the channel number to 64. This is followed by four standard ResNet bottleneck blocks, each comprising two 1 × 1 convolutions and one 3 × 3 convolution, increasing the channel number to 256. The resulting feature map size is 120 × 120 × 256. The second stage introduces two parallel branches with resolutions of 120 × 120 and 60 × 60 and channel numbers of 32 and 64, respectively. Each branch contains four BasicBlocks (each with two 3 × 3 convolutions) for scale-specific feature extraction. Cross-branch fusion modules share multi-scale features. The resulting feature maps are 120 × 120 × 32 and 60 × 60 × 64. In stages 3 and 4, the number of branches expands to three and four, respectively. Each branch continues with four BasicBlocks, and multi-scale fusion is performed at the end of each stage. The feature maps for stage 3 are 120 × 120 × 32, 60 × 60 × 64, and 30 × 30 × 128. For stage 4, they are 120 × 120 × 32, 60 × 60 × 64, 30 × 30 × 128, and 15 × 15 × 256.

#### 2.3.2. Optimization of HRNetV2 Semantic Segmentation Model

**Figure 5:**
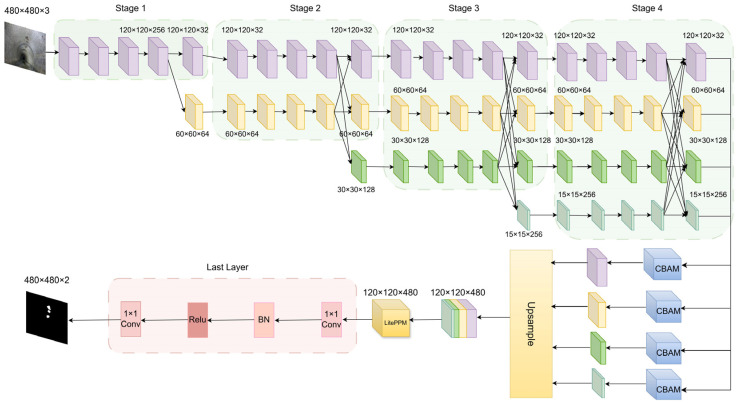
Improved HRNetV2 model.

To further enhance the performance of HRNetV2 in the task of corrosion region segmentation in drainage pipelines, this study introduces structural improvements targeting its limitations in complex environments. Although HRNetV2 possesses a multi-branch parallel structure that maintains high-resolution feature representation, its original design lacks an explicit attention mechanism. As a result, the model struggles to effectively distinguish critical features from redundant background information—particularly under challenges such as blurred corrosion boundaries, unclear textures, and severe background interference. In this study, CBAMs are incorporated into the outputs of the four branches in the stage 4 of the HRNetV2 backbone. The rationale is as follows: First, stage 4 is the final multi-scale feature extraction stage in HRNetV2. Its four branch outputs correspond to different spatial resolutions (120 × 120 × 32, 60 × 60 × 64, 30 × 30 × 128, and 15 × 15 × 256), and the features have already integrated multi-level semantic information. By adding CBAM to stage 4, the dual attention mechanism—channel and spatial—can be fully leveraged to enhance the response of key regions, ensuring that corrosion-related features are emphasized at each scale while suppressing background noise. Second, the CBAMs is lightweight and does not introduce significant parameter overhead, making it suitable for feature enhancement after parallel branches. Moreover, considering that the original HRNetV2 lacks a global contextual modeling mechanism after multi-scale feature fusion—leading to misclassification or omission in the presence of corrosion regions with varying scales and complex shapes—this study introduces a custom-designed LitePPM module after upsampling and channel-wise concatenation of all feature branches. The reasons for placing LitePPM after the fusion step are twofold: First, The fused feature map integrates spatial details and semantic information from multiple scales. Inserting LitePPM at this point enables effective capture of global semantic dependencies, thereby enhancing the model’s ability to perceive large-scale or spatially varying corrosion regions and significantly improving the recall rate. Second, Compared to embedding a context module directly in the backbone, inserting LitePPM here serves a clearer global modeling purpose, contributing to the model’s improved recognition of corrosion areas in complex backgrounds. Finally, the context-enhanced fused features are passed through a 1 × 1 convolution layer for channel compression and then fed into a Softmax classifier to generate binary segmentation results, achieving pixel-wise prediction of the corrosion regions. The improved network architecture is illustrated in Figure 5.

#### 2.3.3. Integration of the CBAM Attention Mechanism

**Figure 6:**
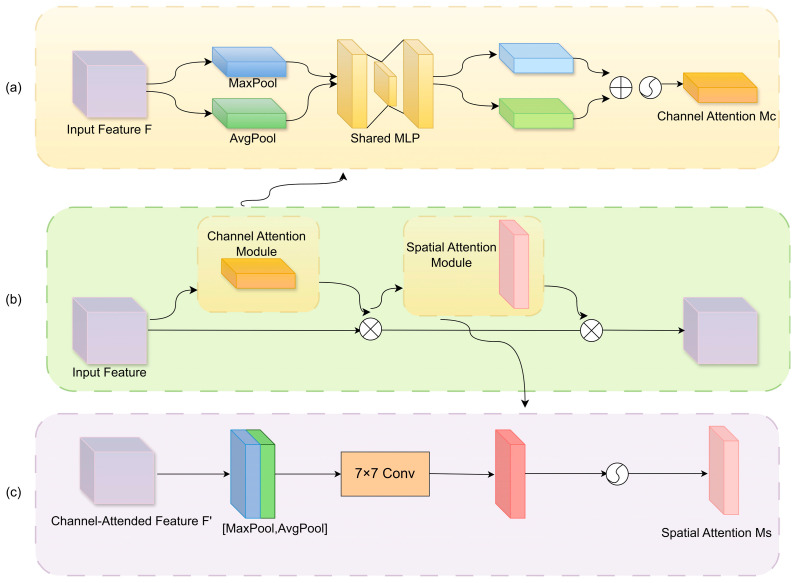
Structure of CBAM: (**a**) Channel attention module; (**b**) CBAM; (**c**) Spatial attention module.

To improve the segmentation accuracy of corroded regions in drainage pipelines, this study integrates the Convolutional Block Attention Module (CBAM). This mechanism aims to address the challenge of weak responses of corrosion features within complex backgrounds, where critical information is often overwhelmed by irrelevant features. CBAM was first introduced by Woo et al. [36] in 2018, and its core idea is to guide the network’s attention towards more informative features by explicitly modeling both channel and spatial attention. As shown in Figure 6, the CBAMs consists of two sequential submodules: the Channel Attention Module (CAM) and the Spatial Attention Module (SAM). The CAM is designed to capture the importance of each feature channel by leveraging both global average pooling and global max pooling, followed by a shared Multi-Layer Perceptron (MLP). The spatial attention module, in contrast, focuses on “where to emphasize by enhancing the network’s sensitivity to critical spatial regions.

The CAM focuses on mining the response differences among channels to generate a channel-wise importance weight map, thereby enhancing the network’s ability to model salient semantic features. Specifically, global average pooling and global max pooling are first applied to the input feature map ${F\in R}^{C\times H\times W}$, resulting in two global context descriptors, $z_{avg\;}$ and $z_{max\;}$. These descriptors are then passed through a shared MLP composed of two fully connected layers with intermediate dimensionality reduction and expansion controlled by a compression ratio r. The outputs are combined and activated using a Sigmoid function to obtain the channel attention weight map $M_{c}$. This weight map is multiplied with the input feature map on a channel-wise basis to perform channel attention recalibration. The computation is expressed as:

(1) $$M_{c}=\sigma \left(W_{2}\times \delta \left(W_{1}\times z_{avg}+W_{1}\times z_{max}\right)\right)$$

where $z_{avg\;=\;GAP\left(F\right)\in R^{C\times 1\times 1},}$ $z_{max\;=\;GMP\left(F\right)\in R^{C\times 1\times 1},}W_{1}=R^{\frac{C}{r}\times C},W_{2}=R^{C\times \frac{C}{r}},$ δ denotes ReLU activation function, σ denotes the Sigmoid function.

The SAM concentrates on “which spatial regions” deserve more attention, thereby improving the model’s ability to discriminate local areas. Taking as input the feature map refined by the channel attention, it first performs average pooling and max pooling operations along the channel dimension, generating two single-channel spatial descriptors. These are concatenated along the channel axis and passed through a convolutional layer with a 7 × 7 kernel, followed by a Sigmoid function to generate the spatial attention map $M_{s}$. Finally, the spatial attention map is multiplied element-wise with the input feature map to enhance spatially relevant locations. The calculation is as follows:

(2) $$M_{s\;}=\sigma \left(f^{7\times 7}\left(\left[AP\left(F\right);\;MP\left(F\right)\right]\right)\right)$$

where ${\;f}^{7\times 7\;}$ represents the convolution operation with a 7 × 7 kernel, AP and MP  denote the average pooling and max pooling along the channel dimension, respectively, and ${\;M}_{s}\in R^{1\times H\times W}$ denotes the Sigmoid function.

#### 2.3.4. Introduction of the LitePPM Module

**Figure 7:**
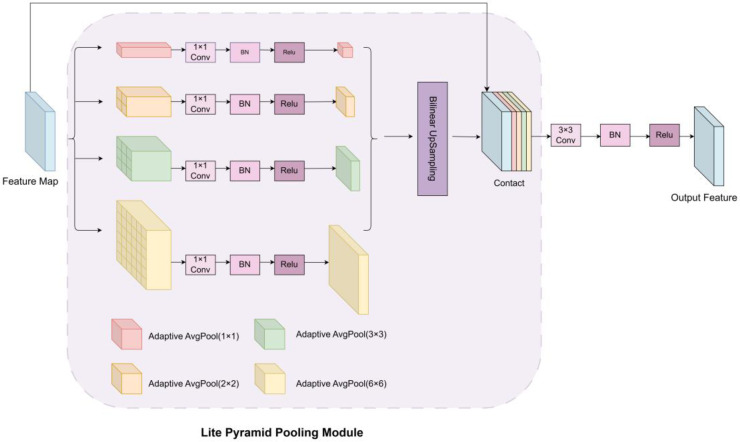
Structure of the LitePPM module.

In complex backgrounds with small targets, traditional segmentation networks often struggle due to limited receptive fields or loss of semantic information at high levels. In real-world pipeline inspections, corrosion areas vary greatly in shape and size, requiring strong contextual awareness. To address this, the LitePPM module is introduced after multi-scale feature fusion. Inspired by the pyramid pooling module in PSPNet [37], LitePPM adopts a lightweight design to provide effective multi-scale context extraction with low parameter overhead. The module includes a multi-scale adaptive pooling stage and an upsampling and fusion stage, as shown in Figure 7.

In the first stage, adaptive average pooling is applied at multiple scales (1 × 1, 2 × 2, 3 × 3, 6 × 6) to extract contextual information with varying receptive fields. Each pooled feature is compressed via a 1 × 1 convolution, normalized using BatchNorm, and activated with ReLU. In the second stage, all features are upsampled using bilinear interpolation to the original size and concatenated with the original feature map. Finally, a 3 × 3 convolution block fuses the concatenated features to produce the enhanced context feature map.

## 3. Experiments and Analysis

### 3.1. Experimental Platform and Parameters

All experiments in this study were conducted under the Windows 10 environment. The hardware configuration includes an Intel(R) Xeon(R) Gold 6133 CPU @ 2.50 GHz processor (Intel Corporation, Santa Clara, CA, USA), 256 GB of RAM, and an NVIDIA GeForce RTX 4090 GPU with 24 GB of memory (NVIDIA Corporation, Santa Clara, CA, USA). CUDA 12.6 was used for parallel acceleration, and the programming language was Python 3.8 with the PyTorch 2.2.0 deep learning framework. During model training, hrnetv2_w32 was selected as the backbone feature extraction network, and the number of output channels was set to 2 for binary segmentation of corrosion and background regions. To ensure fair comparison, all experiments were conducted under the same training settings and hyperparameters, which were carefully tuned to optimize model performance. The final configuration is summarized in Table 2.

### 3.2. Loss Function

To improve performance in segmenting corrosion areas under sample imbalance and enhance boundary precision, a combined loss function is designed. It consists of Dice Similarity Coefficient Loss (Dice Loss) and Focal Loss, equally weighted.

Dice Loss is derived from the Sørensen–Dice coefficient [38] and is commonly used in medical imaging and semantic segmentation. It optimizes overlap similarity and alleviates training bias caused by class imbalance. The Dice Loss is defined in Equation (3):

(3) $$Dice\;Loss=1-\frac{2\sum_{i=1}^{N}p_{i}g_{i}\;+\;\varepsilon }{\sum_{i=1}^{N}p_{i}+\sum_{i=1}^{N}g_{i}+\varepsilon }$$

where $p_{i}\;$ is the predicted pixel value, $g_{i}$ is the ground truth label, N is the number of pixels, and ε is a small constant to prevent division by zero. Dice Loss maximizes the intersection between prediction and ground truth, improving sensitivity to edges and small regions.

Focal Loss, proposed by Lin et al. [39], addresses extreme class imbalance by down-weighting well-classified examples and focusing on hard examples. It is particularly suitable for cases where the target occupies a small area. Equation (4) presents the definition of the Focal Loss:

(4) $$Focal\;Loss=-\alpha {\left(1-p_{t}\right)}^{\gamma }\log\left(p_{t}\right)$$

where $p_{t}\;$ is the predicted probability of the true class, $\alpha \in \left(0,1\right)$ balances class weights, and γ≥0 is the focusing parameter. Typically, γ=2,α=0.25. In this study, γ is set to its default value of 0, class weights are set as 1:1 for foreground and background.

The final loss function is defined in Equation (5):

(5) $$Total\;Loss=\lambda_{1}Dice\;Loss+\lambda_{2}Focal\;Loss$$

where $\lambda_{1}=\lambda_{2}=1$, meaning both losses contribute equally. This combination accelerates convergence and improves boundary discrimination, providing better robustness in detecting blurred edges and low-contrast corrosion regions.

### 3.3. Evaluation Metrics

To comprehensively evaluate the model’s performance on the drainage pipeline corrosion segmentation task, several mainstream semantic segmentation metrics were employed, including Precision, mPrecision, IoU, Accuracy, Recall, F1 and mIoU.

(6) $$Precision=\frac{TP}{TP+FP}$$

(7) $$IoU=\frac{TP}{TP+FP+FN}$$

(8) $$Accuracy=\frac{TP+TN}{TP+TN+FP+FN}$$

(9) $$Recall=\frac{TP}{TP+FN}$$

(10) $$F1=2\times \frac{Precision\times Recall}{Precision+Recall}$$

(11) $$mIoU=\frac{1}{n}\sum_{i=1}^{n}\frac{{TP}_{i}}{{TP}_{i}+{FP}_{i}+{FN}_{i}}$$

where TP denotes the number of pixels correctly predicted as corrosion, FP denotes background pixels incorrectly predicted as corrosion, FN represents corrosion pixels incorrectly predicted as background, and TN represents background pixels correctly predicted as background.

### 3.4. Experimental Results and Analysis

To verify the effectiveness of the proposed improved model in semantic segmentation tasks, we compared its performance with several mainstream models, including DeepLabV3+, PSPNet, and U-Net. All models were trained and evaluated under the same dataset and training strategies. The results are presented in Table 3.

PSPNet and DeepLabV3+ demonstrated excellent performance supported by the lightweight backbone MobileNetV2, achieving mIoU values of 94.62 ± 0.02% and 95.24 ± 0.03%, and Recall values of 96.43 ± 0.02% and 97.02 ± 0.03%, respectively. However, these models exhibited limitations in handling fine structures and boundary regions, with occasional misclassification. U-Net, when combined with ResNet50 and VGG16 as backbones, further enhanced feature extraction capabilities and demonstrated strong accuracy and stability. ResNet50 was preferred as the backbone due to its deep residual structure, which effectively mitigates gradient vanishing and enhances multi-scale semantic expression. When using ResNet50, U-Net achieved mIoU, Recall, Accuracy, Precision, and  F1  of 95.79 ± 0.03%, 96.95 ± 0.04%, 98.49%, 96.32 ± 0.05%, and 96.63 ± 0.03%, respectively. Compared to DeepLabV3+, mIoU improved by 0.58 ± 0.03%, indicating better pixel-level classification accuracy within the target regions. SegFormer achieved mIoU, Recall, Accuracy, Precision, and F1 of 95.1 ± 0.03%, 97.12 ± 0.03%, 98.35 ± 0.02%, 94.85 ± 0.03%, and 95.97 ± 0.02%. HRNetV2, with its high-resolution feature preservation and multi-scale fusion mechanism, achieved superior spatial localization and global contextual awareness. Among HRNetV2 variants, HRNetV2_W32 outperformed HRNetV2_W18 due to its larger number of channels and stronger representational power, with mIoU and Accuracy reaching 95.82 ± 0.03% and 98.51%. After integrating attention mechanisms and context enhancement modules, the improved HRNetV2_W32 model achieved the highest performance: mIoU of 95.92 ± 0.03%, Recall of 97.01 ± 0.02%, Accuracy of 98.54%, Precision of 96.6 ± 0.03% and F1 of 96.8 ± 0.03%, confirming the effectiveness and adaptability of the proposed method.

**Figure 8:**
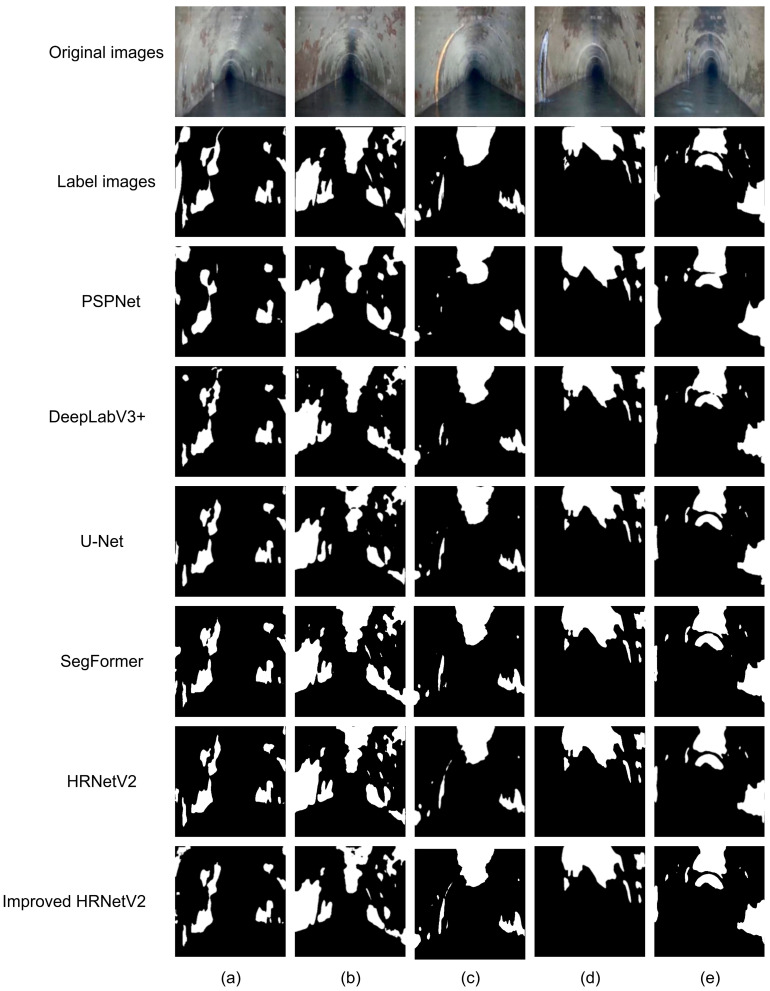
Segmentation results of different models: (**a**) Segmentation results of all models for the first original image; (**b**) Segmentation results of all models for the second original image; (**c**) Segmentation results of all models for the third original image; (**d**) Segmentation results of all models for the fourth original image; (**e**) Segmentation results of all models for the fifth original image, where white regions represent corrosion areas and black regions represent the background.

Figure 8 illustrates the segmentation results of different models. The first row displays the original images, the second row shows the manually annotated ground truth labels, and the third to seventh rows present the segmentation outputs of PSPNet, DeepLabV3+, U-Net, the original HRNetV2, and the proposed improved HRNetV2 model, respectively. From the results, PSPNet demonstrates certain robustness in detecting large-scale corrosion regions, successfully outlining the main contours of the corrosion areas. However, it exhibits significant shortcomings in boundary handling and small-object detection. In Figure 8c, some fine corrosion areas are missed, while Figure 8b shows adhesion issues in adjacent regions. DeepLabV3+ outperforms PSPNet in terms of regional integrity and can more effectively identify medium-sized corrosion areas. However, it still tends to miss or falsely detect regions with blurry edges or small targets. For instance, in Figure 8e, the boundaries of corrosion regions are not clearly defined, and in Figure 8c, the small corrosion regions are not segmented at all. U-Net, with its encoder–decoder architecture, exhibits good continuity and structural recovery in medium-sized corrosion area detection. Nonetheless, its performance in recognizing small corrosion areas, such as in Figure 8c, is suboptimal. It tends to over-smooth features, which negatively impacts boundary precision. In Figure 8a, it also fails to segment corrosion regions with indistinct edges. The original HRNetV2 model, utilizing a parallel multi-branch structure, shows certain advantages in preserving segmentation details and maintaining high-resolution feature extraction. In Figure 8a,c, its output offers clearer edge retention compared to the models mentioned above and demonstrates better detail recovery than U-Net and DeepLabV3+. However, due to the lack of explicit attention mechanisms and contextual information enhancement modules, it still struggles in distinguishing regions under complex texture interference, with some minor corrosion areas—such as those in Figure 8e—being missed. SegFormer sometimes produces over-segmentation in complex pipeline environments, which may lead to false detection, as illustrated in Figure 8b. In contrast, the proposed improved HRNetV2 model achieves the best segmentation results across all samples. Benefiting from the CBAMs, which enhances the perception of key regions, and the LitePPM module, which improves multi-scale contextual modeling, the model can more accurately capture the edges and fine details of corrosion areas. The predictions in the seventh row are highly consistent with the ground truth labels, with the shape, location, and boundaries of corrosion regions being more precisely represented. The model significantly outperforms other comparative methods in both segmentation accuracy and stability.

### 3.5. Ablation, Efficiency, and Visualization Analysis

To further investigate the contribution and focus of different modules in the improved model, ablation studies and Grad-CAM visualizations were conducted to assess feature responsiveness both qualitatively and quantitatively.

In terms of quantitative evaluation, Table 4 shows that the baseline HRNetV2 model achieved IoU of 93.54 ± 0.02%, mIoU of 95.82 ± 0.03%, Recall of 96.87 ± 0.02%, Accuracy of 98.51%, Precision of 96.45 ± 0.02%, and F1 of 96.66 ± 0.02%. After adding the CBAM, the model’s ability to focus on critical areas improved, slightly increasing all metrics. Introducing the LitePPM module alone enhanced context modeling, leading to an mIoU of 95.98 ± 0.02% and F1 of 96.74 ± 0.03%. The combined model with both CBAM and LitePPM achieved mIoU of 95.92 ± 0.03% and F1 of 96.8 ± 0.03%, showing that the two modules are complementary and improve overall performance. It is worth noting that the mIoU of the LitePPM-only variant is slightly higher than that of the improved model. However, the combined model achieves the highest Accuracy and competitive F1, indicating that CBAM contributes to better feature selectivity and robustness, even if a marginal fluctuation in mIoU is observed. Such differences are within the expected variance of repeated training, as confirmed by reporting results across multiple independent runs (mean ± std). Therefore, the complementarity of CBAM and LitePPM should be understood as providing a balanced trade-off across multiple metrics, enhancing the overall stability and generalization of the network rather than solely optimizing a single indicator.

To further evaluate efficiency and lightweight characteristics, Table 5 provides results for model parameters, FLOPs, FPS, and memory usage. When CBAM was combined with LitePPM, the model maintained high segmentation accuracy while keeping computational complexity and efficiency at a reasonable level.

**Figure 9:**
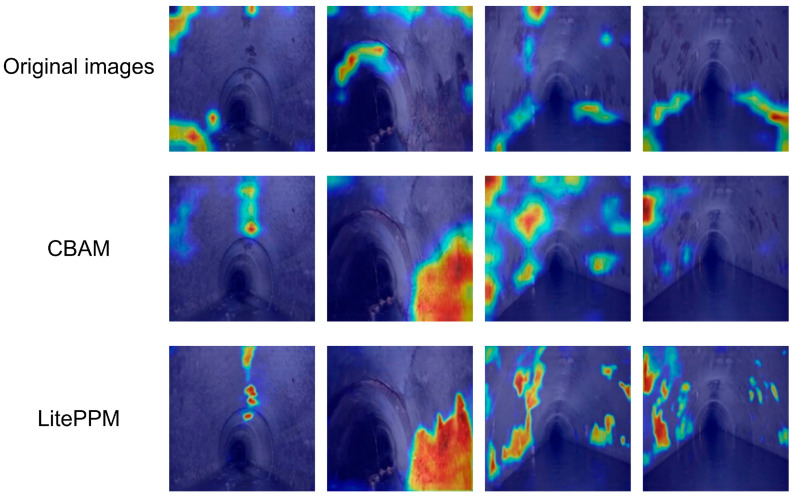
Grad-CAM visualization of attention across different architectures.

For qualitative analysis, Figure 9 presents Grad-CAM visualizations: the first row shows baseline HRNetV2 with scattered and less accurate focus; the second row (with CBAM) shows improved attention to corrosion edges; the third row (with LitePPM) highlights stronger, more continuous focus on corrosion regions, proving its effectiveness in context modeling and localization.

**Figure 10:**
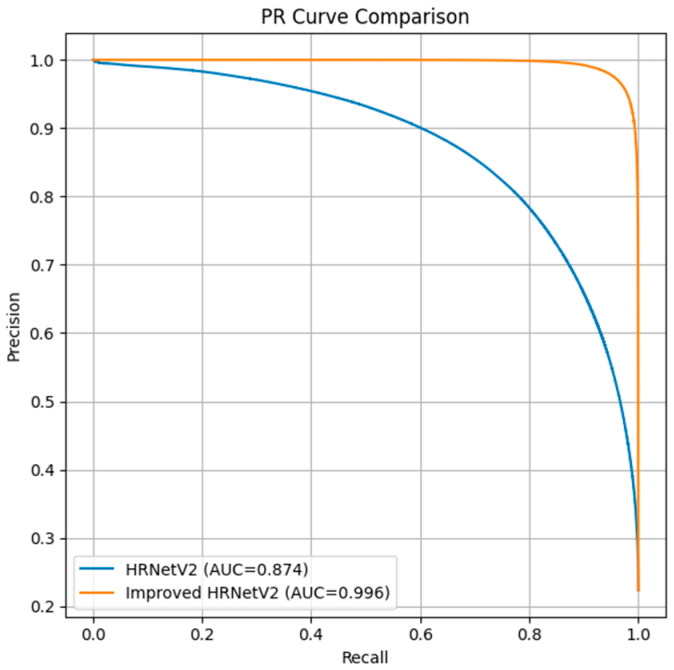
PR Curve.

In addition, Figure 10 presents the Precision–Recall (PR) curves of the baseline HRNetV2 and the improved HRNetV2. The improved model demonstrates a substantially higher area under the curve (AUC = 0.996) than the baseline (AUC = 0.847), confirming its superior trade-off between precision and recall across different thresholds.

## 4. Discussion

This study proposed an improved HRNetV2-based network to address challenges in segmenting corroded regions in drainage pipelines, such as low precision, missed or false detections under complex backgrounds, and limited contextual modeling. By incorporating CBAM into stage 4 outputs, the network was guided to emphasize discriminative features while suppressing background noise. In addition, LitePPM was introduced after multi-scale feature fusion, enabling global context modeling with minimal parameter overhead. The ablation results confirmed that both modules individually improved segmentation accuracy, and their combination achieved the best balance between performance and efficiency.

Beyond accuracy metrics, computational efficiency was also analyzed. Compared to the baseline HRNetV2, the improved model exhibited slightly higher parameter counts but significantly reduced FLOPs and achieved faster inference speed with only marginal GPU memory growth. These findings demonstrate that the LitePPM module not only enhances context representation but also maintains a lightweight design suitable for practical deployment. The CBAM, although adding a negligible parameter overhead, further boosted feature selectivity and interpretability, as evidenced by Grad-CAM visualization, which showed sharper boundary focus and more coherent attention to corrosion regions.

Nevertheless, some limitations remain. The dataset was collected from a single pipeline environment, and generalization to diverse materials, imaging devices, and environmental conditions has not been fully validated. Additionally, this study primarily focused on binary segmentation of corrosion versus background, without extending to multi-class defect detection such as cracks and deformations. Future research could explore larger and more diverse datasets, incorporate boundary-aware metrics (e.g., Boundary IoU, Trimap-F) for finer evaluation, and investigate lightweight deployment strategies for real-time inspection in embedded devices or edge computing platforms.

## 5. Conclusions

In this work, an improved HRNetV2-based semantic segmentation framework was developed for corrosion detection in drainage pipelines. By integrating CBAM and LitePPM modules, the model achieved superior accuracy and efficiency compared to classical segmentation models and the baseline HRNetV2. Specifically, the proposed method attained an  IoU of 93.7 ± 0.02%, mIoU of 95.92 ± 0.03%, Recall of 97.01 ± 0.02%, Accuracy of 98.54%, Precision of 96.6 ± 0.03%, and F1 of 96.8 ± 0.03%, while simultaneously reducing FLOPs and improving inference speed. Grad-CAM visualizations further confirmed that the enhanced model effectively concentrated on corrosion boundaries and regions of interest, improving interpretability and reliability.

Overall, the findings demonstrate that the proposed approach not only improves segmentation accuracy but also offers practical value in supporting the intelligent inspection and maintenance of urban drainage systems. At the same time, we acknowledge that the dataset used in this study was collected within a university campus environment, which may differ from municipal drainage networks in terms of wastewater composition and corrosion patterns. To enhance generalizability, future work will focus on expanding defect categories, conducting cross-site validation under diverse pipeline conditions, and optimizing deployment for real-time applications.

## Figures and Tables

**Table 1:** Description of the dataset.

Item	Description
Total images	1360 images
Image resolution	480 × 480 pixels
Classes defined	Background (0), Corrosion (FS, 1)
Annotation tool	LabelMe (pixel-level annotation)
Data split	Training (80%, 1088), Validation (10%, 136), Testing (10%, 136)
Viewpoints covered	Front views, side views, partial views of pipelines

**Table 2:** Tuned hyperparameters.

Hyperparameter	Value
Epoch	221
Batch Size	5
Optimizer	SGD
Momentum	0.9
Init Learning Rate	4 × 10^−3^
Min Learning Rate	4 × 10^−5^
Learning Rate Decay Type	COS
Weight Decay	1 × 10^−4^

**Table 3:** Evaluation metrics of different models.

Network	Backbone	IoU	mIoU	Recall	Accuracy	Precision	F1
DeepLabv3+	MobileNetV2	92.68 ± 0.03	95.24 ± 0.03	97.02 ± 0.03	98.29 ± 0.02	95.38 ± 0.03	96.19 ± 0.02
PSPNet	MobileNetV2	91.72 ± 0.02	94.62 ± 0.02	96.43 ± 0.02	98.05 ± 0.01	94.93 ± 0.02	95.67 ± 0.02
U-Net	ResNet50	93.5 ± 0.03	95.79 ± 0.03	96.95 ± 0.04	98.49	96.32 ± 0.03	96.63 ± 0.03
U-Net	VGG16	93.41 ± 0.02	95.73 ± 0.02	96.63 ± 0.03	98.48	96.55 ± 0.03	96.59 ± 0.03
SegFormer	B2	92.27 ± 0.03	95.1 ± 0.03	97.12 ± 0.03	98.35 ± 0.02	94.85 ± 0.03	95.97 ± 0.02
HRNetV2	HRNetV2_W18	93.14 ± 0.01	95.56 ± 0.02	96.7 ± 0.03	98.41	96.19 ± 0.04	96.44 ± 0.03
HRNetV2	HRNetV2_W32	93.54 ± 0.02	95.82 ± 0.03	96.87 ± 0.02	98.51	96.45 ± 0.02	96.66 ± 0.02
**Improved HRNetV2**	**HRNetV2_W32**	93.7 ± **0.02**	95.92 ± **0.03**	97.01 ± **0.02**	**98.54**	96.6 ± **0.03**	96.8 ± **0.03**

The results of the improved method are shown in bold.

**Table 4:** Ablation study results.

HRNetV2	CBAM	LitePPM	IoU	mIoU	Recall	Accuracy	Precision	F1
√	×	×	93.54 ± 0.02	95.82 ± 0.03	96.87 ± 0.02	98.51	96.45 ± 0.02	96.66 ± 0.02
√	√	×	93.55 ± 0.02	95.85 ± 0.03	96.92 ± 0.03	98.52	96.5 ± 0.03	96.71 ± 0.03
√	×	√	93.75 ± 0.02	95.98 ± 0.02	96.98 ± 0.03	98.53 ± 0.01	96.5 ± 0.04	96.74 ± 0.03
√	√	√	93.7 ± 0.02	95.92 ± 0.03	97.01 ± 0.02	98.54	96.6 ± 0.03	96.8 ± 0.03

“√” indicates that the module is included in the model, and “×” indicates that the module is not included.

**Table 5:** Model complexity and inference efficiency comparison.

HRNetV2	CBAM	LitePPM	Params (M)	FLOPs (G)	GPU Memory (MB)	FPS (Images/s)
√	×	×	29.53	39.96	1112.77	5.83
√	√	×	29.55	39.60	1115.97	5.89
√	×	√	30.35	37.67	1117.82	6.31
√	√	√	31.41	35.92	1119.57	7.21

“√” indicates that the module is included in the model, and “×” indicates that the module is not included.

## Data Availability

The dataset used in this study originates from the drainage pipeline corrosion images captured by a CCTV inspection robot at China University of Mining & Technology, Beijing. The authors have obtained permission to use the full dataset. To facilitate reproducibility and external validation, a subset of the dataset, including raw images and their corresponding ground truth masks, has been made publicly available at https://github.com/Planet82/sewer-datasets (accessed on 11 September 2025). The complete dataset can be accessed upon reasonable request by contacting the corresponding author. This public release is intended to encourage further research and facilitate benchmarking in sewer defect detection tasks.

## Abbreviations

The following abbreviations are used in this manuscript:

HRNetV2	High-Resolution Network Version 2
CBAM	Convolutional Block Attention Module
LitePPM	Lightweight Pyramid Pooling Module
mIoU	mean Intersection over Union
CCTV	Traditional closed-circuit television
CNNs	Convolutional Neural Networks
U-Net	U-shaped convolutional networks
CA	Coordinate Attention
SE	Squeeze-and-Excitation
ASPP	Atrous Spatial Pyramid Pooling
ECA	Efficient Channel Attention
CRF	Conditional Random Field
UAV	Unmanned Aerial Vehicle
StyleGAN3	Style-Based Generative Adversarial Network 3
ResNet	Residual Network
PSPNet	Pyramid Scene Parsing Network
HRNet	High-Resolution Network
HRNetV1	High-Resolution Network Version 1
HRNetV2P	High-Resolution Network Version 2 Plus
SAM	Spatial Attention Module
CAM	Channel Attention Module
MLP	Multi-Layer Perceptron
Dice Loss	Dice Similarity Coefficient Loss
